# Sharing is caring: a call for a new era of rare disease research and development

**DOI:** 10.1186/s13023-022-02529-w

**Published:** 2022-10-27

**Authors:** Nathan Denton, Andrew E. Mulberg, Monique Molloy, Samantha Charleston, David C. Fajgenbaum, Eric D. Marsh, Paul Howard

**Affiliations:** 1 Gene Therapy Program, Department of Medicine, Perelman School of Medicine, University of Pennsylvania, Philadelphia, PA 19104 USA; 2 Neurogene Inc, New York, NY USA; 3 Department of Medicine, Orphan Disease Center, Perelman School of Medicine, University of Pennsylvania, Philadelphia, PA 19104 USA; 4 Translational Medicine & Human Genetics, Perelman School of Medicine, University of Pennsylvania, Pennsylvania, PA 19104 USA; 5 Departments of Neurology and Pediatrics, Perelman School of Medicine, Children’s Hospital of Philadelphia, University of Pennsylvania, Philadelphia, PA 19104 USA; 6 Division of Neurology, Children’s Hospital of Philadelphia, Philadelphia, PA 19104 USA; 7 Amicus Therapeutics, Philadelphia, PA 19104 USA

**Keywords:** Rare diseases, Drug development, Knowledge bases, Registries, Research design, Data management, Clinical trials

## Abstract

Scientific advances in the understanding of the genetics and mechanisms of many rare diseases with previously unknown etiologies are inspiring optimism in the patient, clinical, and research communities and there is hope that disease-specific treatments are on the way. However, the rare disease community has reached a critical point in which its increasingly fragmented structure and operating models are threatening its ability to harness the full potential of advancing genomic and computational technologies. Changes are therefore needed to overcome these issues plaguing many rare diseases while also supporting economically viable therapy development. In “Data silos are undermining drug development and failing rare disease patients (*Orphanet Journal of Rare Disease*, Apr 2021),” we outlined many of the broad issues underpinning the increasingly fragmented and siloed nature of the rare disease space, as well as how the issues encountered by this community are representative of biomedical research more generally. Here, we propose several initiatives for key stakeholders - including regulators, private and public foundations, and research institutions - to reorient the rare disease ecosystem and its incentives in a way that we believe would cultivate and accelerate innovation. Specifically, we propose supporting non-proprietary patient registries, greater data standardization, global regulatory harmonization, and new business models that encourage data sharing and research collaboration as the default mode. Leadership needs to be integrated across sectors to drive meaningful change between patients, industry, sponsors, and academic medical centers. To transform the research and development landscape and unlock its vast healthcare, economic, and scientific potential for rare disease patients, a new model is ultimately the goal for all.

## Background

This position statement aims to encourage meaningful dialogue on the issues of data standardization and sharing between key stakeholders in the rare disease space, principally patients, industry, sponsors, and academic medical centers. We previously outlined many of the broad issues underpinning the fragmentation and siloed nature of the rare disease space, as well as how these issues are not unique to this community but biomedical research more generally [1]. We and many others [2–6] firmly believe that unlocking the full therapeutic/curative and economic potential of the rare disease space requires supporting non-proprietary patient registries [7, 8], greater data standardization [9, 10], global regulatory harmonization [5, 11], and new business models [12, 13] that encourage data sharing and research collaboration as the default mode [14, 15]. Here, we propose several initiatives for key stakeholders to reorient the rare disease space and its incentives in a way that we believe would cultivate and accelerate innovation.

## Main text

The rare disease research community is experiencing an explosion in activity as our understanding has grown for many clinically described disorders with previously unknown etiologies, genetics, and mechanisms [16]. The emergence of improved mechanistic understanding, combined with new tools like gene therapy [17], gene editing [18], and next-generation sequencing [19], has inspired optimism in the patient, clinical, and research communities that disease-specific treatments are on the way. Growing research efforts accompany the notable success of several gene therapy programs to characterize the basic biology and clinical manifestations of rare diseases, with translational studies bridging this crucial gap [20].

Unfortunately, the successes are considerably outnumbered by the failed attempts to develop treatments for rare diseases. Indeed, more than 90% of all rare diseases do not have an approved treatment [21]. Drug development for this community faces multiple challenges, with most issues stemming from the fact that rare diseases affect small patient populations in a non-uniform manner [22]. These small, complex patient populations are often incompatible with many key design and statistical power principles required for traditional randomized controlled trials (RCTs) [23]. This general lack of information (in which available data are often heterogeneous and complex), combined with significant inter- and intra-patient variability (in terms of disease onset, presentation, progression, response to treatment, etc.), results in a high degree of unexplained variation that makes it difficult for researchers and regulators to confidently assess potential efficacy signals.

Registries and natural history studies are potentially well positioned to characterize inter- and intra-patient variability and inform precision-guided trials to test targeted therapeutics in stratified populations. However, many impediments prevent the optimization of these studies in the rare disease context such as poor data collection and data management that is perpetuated by longstanding “gaps in standardization, disparate privacy laws and international regulations, and the shortage of shared international regulatory endorsements of best practices for data collection.” [6] Better tools for accelerated development of rare disease therapeutics will continue to elude us so long as we remain dependent on building blocks (i.e., patient registry and natural history data sources) that are incomplete, flawed, or difficult to use.

That the field is not better organized should be unsurprising to many. Data collected by academic, pharmaceutical, and patient groups on rare disease patients are primarily locked away within proprietary databases that are often selectively leveraged to protect funding streams, as well as support publications and nascent intellectual property. Each of these goals is valid and may not impede rapid advances in more common disease states, like oncology and cardiovascular disease. In the rare disease context, however, this situation typically means that no individual stakeholder can accumulate a critical mass of knowledge to de-risk their rare disease drug development programs in favor of success in the vast number of cases. This thereby delays or denies highly vulnerable patients potential treatments while also promoting the waste of precious time, energy, and resources. Extrapolating current drug development timelines, failure rates, and limited funding streams to the >7,000 known rare diseases suggests that it would take many decades to develop treatments/cures for even a fraction of them [24]. Given the substantial yet oft-underestimated scale of the rare disease burden [22] and immense, growing healthcare costs [25], creating scalable platforms and common methods to leverage our knowledge of shared etiology and pathology across rare diseases would provide the greatest positive impact on this patient population [26].

We have arrived at this unfavorable situation by the key stakeholders merely operating within the current system of economic and academic incentives regarding our most scarce resource: rare disease patient data. The reluctance to develop new standards and incentives to actively encourage data sharing makes the already daunting task of developing rare disease therapies even more difficult, if not self-defeating. There is clear evidence that data sharing and collaboration are highly effective and efficient in improving health [27, 28] and creating value [14, 29] while also enhancing affordability [30]. Moreover, the current situation concerning the proprietary nature of patient registry data goes against the consensus of the community whom the various stakeholders are supposed to serve – the rare disease community – who overwhelmingly wish for their data to be shared (and protected) [7, 31]. This is particularly pertinent given that patients and their families can find it increasingly difficult to participate in rare disease research due to misaligned priorities between stakeholders and a general lack of transparency and collaboration, despite being an extremely motivated and altruistic group who wish to advance science to benefit the community [32, 33]. We are unwitting agents in a system that is a means to an end, but it is unclear for whom and for what.

There is an immense moral imperative to rectify this situation rapidly, with the urgency being two-fold. In addition to rare disease patients’ and their family’s race against time, we are on the cusp of massive data proliferation. Technology and open data rules are democratizing data aggregation and registry formation such that any patient community can easily become data enabled. This looming explosion of registries in the current system is poised to exacerbate data loss, replication, and/or data gaps unless meaningful steps are taken [10]. In a field where high-quality data are already limited, not addressing this avoidable systemic flaw will ensure a rare disease diagnosis will continue to be a tragic and hopeless moment for many patients and their families. Failing to harness the explosion in data collection and analysis techniques will allow this unique, potentially revolutionary, window of opportunity to close and instead leave the field continuing along its path to become simultaneously fragmented yet swamped by a cacophony of largely unusable registries [9].

We believe that the convergence of timing and technology provides a unique and important opportunity for sponsors, patients, and providers to focus on core data use issues - integrity, curation, and interoperability – to help bridge clinical care and clinical research. While we can and should be agnostic about technology standards and platforms, we as a community must adopt foundational principles that enable and encourage the creation of informative patient registries based on FAIR data principles – Findable, Accessible, Interoperable, Reusable [34].

For example, interoperable patient registries or natural history studies can inform strategies for stratification in a clinical trial (e.g., genotype) by helping to identify and test innovative clinical endpoints and outcomes, as well as determine branch points for adaptive trial design. One of the article’s authors can also attest to patient-submitted electronic medical record data and biospecimens providing rich additional detail in real-time to bridge gaps in clinical trial data or guide novel biomarker hypothesis. The community has similarly called for greater guidance from regulators to ensure (adaptive) trials advance as swiftly as possible by utilizing patient- and caregiver-reported outcomes such as seizure frequency, experience of side effects, etc. to assess priors or provide external control arms [26].

This situation - and many others besides - also provides a prime opportunity to devise and deploy better methods to enhance our understanding of the rare disease patient journey and the basic etiology of rare diseases [35] as well as ensure alignment between stakeholders [36] on assessing meaningful outcomes to guide clinical care pathways and product reimbursement. Many of these innovative trial designs and regulatory frameworks based on the agile use of patient data have proven to be viable after their rapid deployment and assessment in response to the COVID-19 pandemic. Despite setbacks in some fields (e.g., oncology [37]), greater use of platform trials, remote and decentralized trials to protect medically fragile patients, and regulators’ growing acceptance of real-world data (RWD) that may provide real-world evidence (RWE) to support both safety and efficacy assessments have enabled biomedical research to continue during the COVID-19 pandemic while being responsive to emerging medical challenges [38]. Generating and integrating FAIR data in the rare disease space will enable more innovative clinical trial design and execution to occur, for this space to evolve more quickly, and for the value of different data types to be determined at different stages to optimize the use of limited resources, time, and energy.

Before the COVID-19 pandemic, a handful of cases operating within the constraints of the current research and development ecosystem successfully implemented a ‘next-generation’ approach in their use of patient data. They were able to develop comprehensive datasets, accelerate understanding and innovation, and ultimately devise and evaluate effective interventions in a relatively short amount of time. For example, the Castleman Disease Collaborative Network combined pre-existing clinical data with proteomic data, machine learning, and statistics to compile and analyze a dataset containing fewer than 100 patients; from this, it was possible to create a molecular subtyping method that resulted in the delineation of distinct pathogenic mechanisms [39] that enabled the development of an effective treatment regimen [40]. A foundational aspect of this effort was the ACCELERATE natural history registry, which combined patient medical record data with patient-reported outcomes data and biospecimens from patients. This example also highlights the potential for drug repurposing as the new treatment regimen discovered through proteomics was a pre-existing cancer treatment that was repurposed for Castleman disease [41]. Significant improvements to patients’ lives are evidently attainable by collating current resources - however modest – and engaging in collaborative efforts to bridge gaps. As rare disease research advances, the benefits of combining omics with clinical phenotype data/biological samples [42] and conducting multi-omic research grow increasingly clear in various contexts [43], from obtaining diagnoses to the molecular characterization of disease and its subtypes to identifying disease biomarkers and novel therapeutic targets [44, 45]. Realizing these benefits, however, is contingent on a collaborative culture and standardization measures that are yet to fully manifest.

The Critical Path Institute’s Rare Disease Cures Accelerator Data Analytics Platform (RDCA, supported by the U.S. FDA and NORD) and RARE-X also represent forward-thinking platforms focused on aggregating, curating, and integrating datasets to improve the rare disease research and development ecosystem. These ventures provide a window into the advantages and possibilities enabled by standards-based data sharing, aggregation, and analysis to inform study design (e.g., to validate novel endpoints) and prospective data collection (including patient-level data) in the rare disease space to support both regulatory and post-market use cases (including clinical care and outcomes-based contracting); they also provide a model of transparent, inclusive, and patient-empowering data governance practices through industry and regulatory engagement. Although these initiatives provide grounds for hope, it must be emphasized that they are still the exception, rather than the norm.

Groups at the NIH and FDA have started programs to address the issues affecting the rare disease field, but we and many others feel that there is a general lack of clarity and direction from regulators on data sharing and data standards to support regulatory decision making. With this in mind, we organized a webinar in September 2020 entitled “Let’s Get Real: Harnessing Non-Proprietary Patient Registries and RWE to Accelerate Rare Disease Drug Development.” [46] This series began a much-needed discussion of issues regarding regulatory guidance/best practices, data sharing, and novel approaches to natural history studies and the use of real-world data. Panelists from industry, the FDA, the NIH, academia, and patient foundations all contributed to the webinar series, which involved public sessions open to the community and closed whiteboarding sessions that explored the ideas and questions raised in each session in greater depth.

The last webinar presented a dialogue with Dr. Amy Abernethy, former Principal Deputy Commissioner of the FDA, on the agency’s approach to real-world data, and the lessons learned from the COVID-19 pandemic. After these discussions, we wholeheartedly believe that it is possible to challenge current assumptions and to positively disrupt the research ecosystem by carefully deploying policies and incentives to effect mutually beneficial change. It is abundantly clear that any stakeholder acting alone cannot tackle the challenges facing the rare disease space. Moreover, there is no single solution to this complex and evolving situation as the underlying technologies and analytical tools change over time. However, common guiding principles, agreement on best data practices/standards, and reusable infrastructure employed by the various national and international stakeholders could bring about significant positive change rapidly within the rare disease ecosystem. We overview the key problems and propose solutions in the following sections.

### Problem 1

Small, highly geographically dispersed patient populations across multiple regulatory jurisdictions make it extremely difficult to aggregate enough data to advance innovative tools for rare disease drug development.

### Proposed solution

Build national and international consensus among stakeholders - guided by regulatory leadership - to determine high-quality disease definitions and standardize clinical outcome assessments for individual diseases using Clinical Data Interchange Standards Consortium (CDISC) terminology and standards in anticipation of mapping, sharing, and harmonizing pre-existing datasets.

- Regulatory agencies - such as the FDA and EMA - should embrace the unique role of regulator, technical expert, and convenor/facilitator. They should provide regular forums (both in person and virtual) for discussion and engagement between the different stakeholders (i.e., patient groups, clinical, academic, industry, and regulators). The FDA’s current Patient Focused Drug Development meetings [47] and/or Patient Listening Session [48] format could be used to reach consensus between stakeholders on priority research questions, terminology, data types and their uses (e.g., what constitutes ‘regulatory-grade’ data [49], dataset linkage mechanisms, and analytical methods, including endpoints and adaptive clinical trial design) that ultimately satisfy regulatory standards and span multiple disease groups. Best practices and guidelines should be determined according to successful, transparent collaborations between patient organizations and clinical research sponsors [50] (e.g., community advisory boards [51] and patient advocacy groups [52, 53]) and the academic communities at large (see the FAIR principles – Findable, Accessible, Interoperable, Reusable [34] – to guide the flexible (re)design of current and future registries). High-level research networks, such as the federation of children’s hospitals, could represent key sites to run pilot schemes to hone and standardize processes before broader roll out.
- Align international stakeholders to generate a ‘target data manifest’ for a given rare disease to support therapy development, in which the necessary data types and source(s) are determined for each stage and for what purpose. Data collection methods should be devised around the limitations of data sources to obtain a comprehensive longitudinal data landscape that can inform clinical decision-making and generate a knowledge base that is “fit for purpose” [54].
- Engage with the technology industry to develop systems that utilize common data ontologies to capture and share regulatory-grade data with minimal burden from any/all contributors (and confer the appropriate privacy protections). Regulators should act as a convener for developing common outcomes measures that include algorithms, wearable devices, medical imaging, etc., to create standardized, freely accessible tools which enable better quantitative assessment of core clinical concepts of interest (particularly for common outcomes/symptoms such as seizures/tremors). Utilize technology and flexible/adaptive strategies to optimize research design with a specific rare disease case to reduce wastage, improve efficiency, and maximize effectiveness [55]. Build connections among previously fragmented datasets and support the development of artificial intelligence (AI) and machine learning methods to enhance data analysis [56]. Enable patients to enroll into prospective natural history studies virtually without having to travel to a study site, and task central institutions with obtaining and entering data for each patient (see ACCELERATE for Castleman Disease [41]).

### Problem 2

The general absence of a cultural expectation of pre-competitive data sharing by key stakeholders has led the field to a situation in which data hoarding is the norm. This stifles innovation by making it difficult to develop novel drug development tools, as has been observed in more common therapeutic areas like oncology.

### Potential solution

Stakeholders in industry, academia, and patient groups should be educated about the benefits of data sharing and resource pooling, with a particular focus on key gatekeepers such as children’s hospitals, research director networks, RD CEOs, and patient group leaders. Academics and the NIH should reward responsible data sharing through prioritization of grants, tenure, and publications.

- Engage industry, academia (including journals [57]), patients, and regulators in dialogue so all stakeholders become more comfortable in collecting and sharing medical data in new ways to encourage the voluntary adoption of common data models and best practices [58, 59]. Umbrella rare disease associations should take the lead by providing educational resources and a framework for grading how well organizations achieve FAIR data practices in patient registries and NIH-funded research. These organizations should also lead discussion of how strategies such as federated data exchanges [60] protect privacy and ownership of data. Pharmacosafety and/or pharmacovigilance may represent topic areas which could unify stakeholders and act as a starting point to develop data-sharing practices.
- Increase public funding by national (e.g., countries) and transnational (e.g., European Union, World Health Organization) entities for rare disease research to favor the generation of fewer centralized registries by covering the costs of data curation and data sharing assessments. Such a publicly funded central data infrastructure should support the deposition and dissemination of well-annotated data in formats that enable use by multiple groups. This would help ensure registries are adequately equipped to meet regulator, sponsor, and payer needs while dissembling the proprietary data siloes that have developed due to few major public (e.g., NIH) initiatives for rare diseases [61] coupled with a reliance on public fundraising from families. Targeting funding towards data-enabled registries, through platforms like the RDCA, for example, could enhance the value of future and existing registries by encouraging and supporting the development of biobanks linked to registry entries (in which sample collection, bioassay usage, etc. are standardized and shared). High- quality registry data linked to biospecimens could incentivize additional rare disease research from companies and venture capital by de-risking early-stage proof-of-concept trials. Encourage and support international collaboration (possibly even the merging) between rare disease registries.
- Encourage universities around the world to uncouple the academic promotion/tenure process from data ownership and outcomes that flow almost exclusively from it in favor of data-sharing practices and collective achievements (e.g., formal recognition of research productivity not just via manuscript authorship, but as a data contributor or analyst, in which frequency of citations, data reuse, and/or impact of data analysis, for example, could be included in assessments).

### Problem 3

Existing incentives discourage companies from engaging in more pre-competitive data-sharing platforms because of a lack of clear business models to reward sharing and insufficient incentives and/or pressure to use data responsibly.

### Proposed solution

Policymakers and regulators should define clear use cases for how aggregated, high-quality data sets can be used to satisfy pre- and post-market regulatory requirements, for instance, for Phase IV trials, externally controlled trials, and label expansion.

- Regulatory agencies (e.g., the EMA and FDA) should lead the way in defining use cases for developing stage-appropriate and innovative endpoints [62] that encourage data standardization and data sharing. This could involve providing guidance on how high-quality data from registries and real-world evidence can satisfy post-market requirements for confirmatory trials and create externally controlled trials. Regulators should support the development of a collaborative, non-proprietary/pre-competitive ‘data space’ that encourages data sharing, collaboration, and data curation to support endpoint development (see the FDA’s National Evaluation System for Health Technology [63] and Federal Health IT Strategic Health Plan 2020–2025 [64]).
- Use regulator-hosted forums to build familiarity between stakeholders to conduct innovative regulatory work in real-time to facilitate the meeting of objectives in rare disease research and development. For instance, such forums might be used to identify novel data modalities that enhance understanding and are acceptable for regulatory decision making; this could include patient-entered digital signals, FDA-approved device data, or other datasets (e.g., electronic health records/EHR and insurance claims). These forums should also provide greater representation of patient groups’ perspectives to ensure realistic, feasible, and appropriate standards are being applied consistently to the drug development/approval process in a disease-specific manner. They could also reduce industry’s perception of regulatory risk and create clear value for participants to share “lessons learned” (see FDA’s patient listening sessions [48]) to accelerate the development process.
- Draw upon Congress or equivalent national governments to empower regulators with expert and patient input that encourages and/or enforces data sharing. In a transitory period, Congress could incentivize data-sharing standards by making the training of AI algorithms to support data integration a national strategic priority (e.g., for electronic health records). The FDA could also be empowered to fast-track RWE applications that incorporate data-sharing practices before mandating future applications adhere to data sharing criteria ([59], see Sect. 309).
- Empower patients to be involved in data governance [10] and promote data literacy among patient groups. Develop a policy framework that aspires towards maximal transparency in data usage during sharing (e.g., provide public information on data accession and utilization, akin to the SWIFT system for transactions in banking). Develop and implement best practices for obtaining patient consent in research settings that support patient recruitment and facilitate their role as data generators while protecting their privacy, rights, and well-being without constraining research [65, 66]. Ensure there is a framework in place to ensure that when people “leave their legacy in data,” the patient’s intent is honored and their data are used as effectively as possible and shared as widely as necessary (i.e., in accordance to the FAIR principles) instead of being hoarded.

## Conclusion

The rare disease world is entering a potentially irrevocable state that will exacerbate already-significant delays and obstacles to making advances for rare disease patients in whom permanent loss of function or mortality can be measured in mere months. Given that many rare diseases affect children, meaningful change in this domain is desperately required and massively overdue to support this vulnerable population better. The advent of potentially curative genomic technologies, along with advances in computing power and analytics, provides an opportune time to start a broad, international dialogue and reach consensus between stakeholders on key issues relating to registry design, data sharing, and data governance. At best, the rare disease space will continue to grow and innovate, but this growth will be limited in trajectory, scope, and success. At worst, current structural flaws and practices left unaddressed have the potential to fragment the rare disease research and development landscape permanently, locking the stakeholders in a self-motivated yet counterproductive struggle that stifles innovation and ultimately seals the fate of the vulnerable patients they are supposed to serve.

The necessary transformation of the research and development ecosystem, with its economic and academic incentives that precipitated the current predicament, requires leadership to be shown and dialogue to be started between patients, industry, sponsors, and academia. Regulators need to provide leadership and directionality to the various stakeholders to cultivate an international environment in which top-down guidance (e.g., for regulatory-grade data collection and clear use cases) synergizes with bottom-up innovation (e.g., new business models founded on agile data curation, aggregation, and analytics according to patient-centered data control) to accelerate drug development. This guidance needs to be aligned globally and encouraged through a mixture of novel economic incentives and policies to shift the research ecosystem towards non-proprietary, shared patient registries as the default standard. This scenario would help usher in a new data ecosystem that rewards collaborators for generating regulatory-quality data and analytics that capitalize on the potential of new approaches like AI and machine learning, platform trials, adaptive and remotely conducted trial designs, and real-world data (e.g., from wearable devices) to accelerate rare disease drug development. The solutions are within our grasp. We hope that the various stakeholders will step up to the challenge to maximize the limited resources, time, and energy available in the rare disease world for the sake of the patients and their families.

## Data Availability

Not applicable.

## List of abbreviations

AI: Artificial intelligence.
CDISC: Clinical Data Interchange Standards Consortium.
COVID-19: Coronavirus disease of 2019.
EHR: Electronic health record.
EMA: European Medicines Agency.
FAIR: Findable, Accessible, Interoperable, Reusable.
FDA: Food & Drug Administration.
FOCR: Friends of Cancer Research.
IT: Information technology.
NIH: National Institutes of Health.
NORD: National Organization for Rare Disorders.
ODC: University of Pennsylvania Orphan Disease Center.
RCT: Randomized controlled trial.
RDCA: Rare Disease Cures Accelerator.
RD CEO: Research Director-Chief Executive Officer.
RDCA-DAP: Rare Disease Cures Accelerator-Data and Analytics Platform.
RWD: Real-world data.
RWE: Real-world evidence.
SWIFT: Society for Worldwide Interbank Financial Telecommunication.
