# Correction to: Sharing is caring: a call for a new era of rare disease research and development

**DOI:** 10.1186/s13023-023-02613-9

**Published:** 2023-01-23

**Authors:** Nathan Denton, Andrew E. Mulberg, Monique Molloy, Samantha Charleston, David C. Fajgenbaum, Eric D. Marsh, Paul Howard

**Affiliations:** 1 Gene Therapy Program, Department of Medicine, Perelman School of Medicine, University of Pennsylvania, Philadelphia, PA 19104 USA; 2 Neurogene Inc., New York, NY USA; 3 Department of Medicine, Orphan Disease Center, Perelman School of Medicine, University of Pennsylvania, Philadelphia, PA 19104 USA; 4 Translational Medicine & Human Genetics, Perelman School of Medicine, University of Pennsylvania, Philadelphia, PA 19104 USA; 5 Departments of Neurology and Pediatrics, Children’s Hospital of Philadelphia, Perelman School of Medicine, University of Pennsylvania, Philadelphia, PA 19104 USA; 6 Division of Neurology, Children’s Hospital of Philadelphia, Philadelphia, PA 19104 USA; 7 Amicus Therapeutics, Philadelphia, PA 19104 USA

**Correction to: Orphanet Journal of Rare Diseases (2022) 17:389** 10.1186/s13023-022-02529-w

Following publication of the original article [1], we have been notified that authors Nathan Denton and Eric D. Marsh have incorrect affiliations mentioned in the published articles. The correct affiliations should be as follows:

Nathan Denton*1

Eric D. Marsh*3,5,6
